# Gene variants in periventricular nodular heterotopia

**DOI:** 10.1186/s42494-026-00243-9

**Published:** 2026-05-02

**Authors:** Jianping Song, Xiaoqin Sun, Chunqing Zhang

**Affiliations:** Department of Neurosurgery, Epilepsy Research Center of PLA, Xinqiao Hospital, Army Medical University, 183 Xinqiao Main Street, Shapingba District, Chongqing, 400037 China

**Keywords:** Gene variant, Gray matter heterotopia, Malformation of cortical development, Neuronal migration, Periventricular nodular heterotopia

## Abstract

Periventricular nodular heterotopia (PVNH) is a malformation of cortical development (MCD) mainly caused by aberrant neuronal migration, and a group of diseases sharing similar pathological manifestations, including the presence of nodular clusters of abnormal neurons in the subependymal region. PVNH is one of major causes that result in genetic epilepsy. Seizures can strike as early as a few days after birth but are more common at 10–20 years old, and among them, generalized tonic–clonic seizures are commonly observed. PVNH is a highly genetically heterogeneous disease associated with various rare single gene variants. However, despite the fact that the *FLNA* gene is identified to be closely correlated with the presence of PVNH, mutations in other genes were understudied and have not attracted as much attention due to the relatively low morbidity of PVNH. In consequence, an updated spectrum of PVNH-associated risk genes with potentially pathogenic changes that lead to PVNH in human patients is urgently needed. The risk genes that have already been clinically reported for PVNH are summarized here chronologically according to when the first patient was reported, and clinical manifestations of patients with each of these genes are described. Human cerebral organoids as well as animal models are subsequently discussed in this review to reveal alterations in risk gene products and the pathogenesis of PVNH.

## Highlights

1. This review updates a spectrum of PVNH-associated risk genes that have been clinically reported.
2. General distribution of periventricular nodules seen on imaging by each risk gene mutation is classified here with unified standard.
3. Potentially pathogenic causes of abnormal neuronal migration leading to heterotopia have been increasingly studied.

## Background

The construction of the cerebral cortex is a complicated and elaborate process coordinating progenitor proliferation, neurogenesis, and neuronal migration during embryogenesis [1]. After the early stage of neural tube closure, neuroepithelial cells (NECs) in the ventricular zone (VZ), differentiate into radial glial cells (RGCs) that exhibit polarization with an apical-basal orientation and characteristics of both astrocytes and NECs. RGCs have long basal and short apical processes, establishing a scaffold for neuronal migration through the intermediate zone (IZ) to the developing cortical plate (CP), and forming apical anchors along the ventricular lining for the primary cilium and centrosomes. Most neurons arise from RGCs directly or indirectly [2]. Asymmetric cell divisions of RGCs generate neurons and intermediate progenitors (IPCs) or basal radial glial cells (bRGs) that form the subventricular zone (SVZ) and produce postmitotic neurons. These multipolar neurons migrate radially along the RGC scaffold and become bipolar and are destined for proper layers of the cortex.

**Fig. 1:**
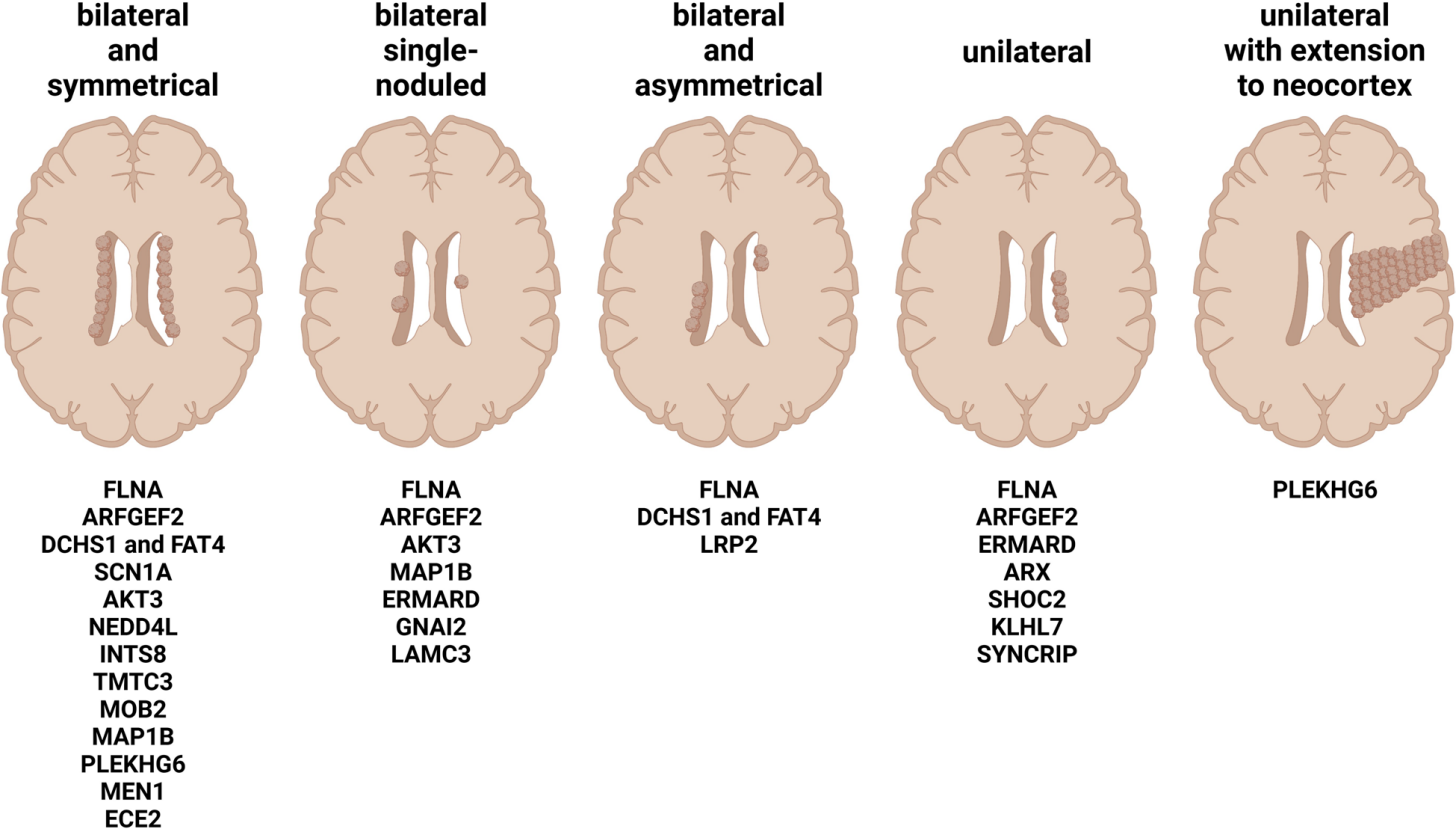
The schematic representation of aberrant neuronal migration and the formation of PVNH nodules

**Fig. 2:**
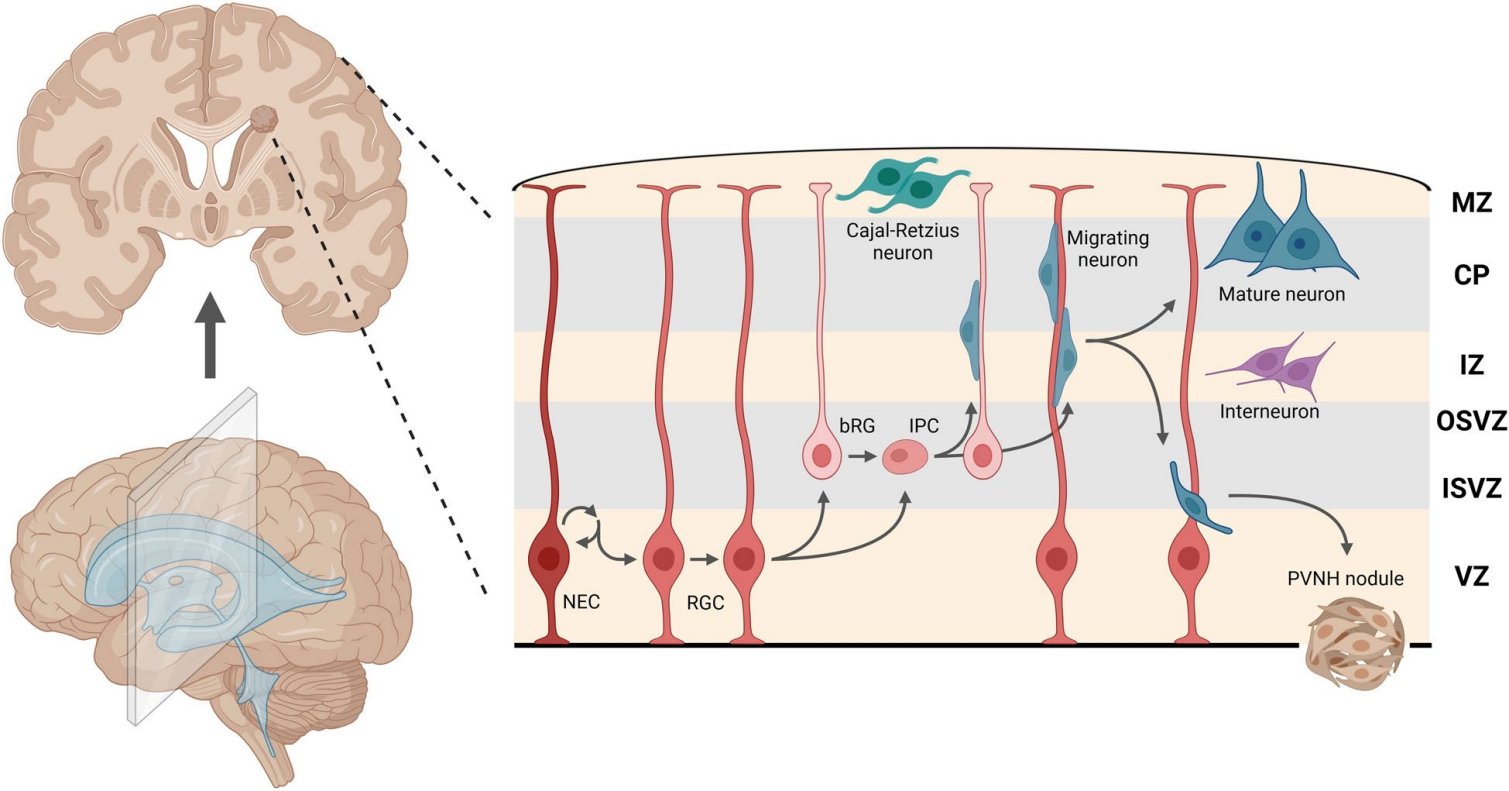
The schematic representation of five types of PVNH according to distribution of nodules, and the list of related risk genes for each type

Malformation of cortical development (MCD) is defined as a broad range of developmental disorders. It includes gray matter heterotopia (GBH) [3], a class of diseases caused by neuronal migration block, and periventricular nodular heterotopia (PVNH) is a clinical subtype of GBH that is characterized by breaks in the integrity of ventricular lining, with some neurons gathering in nodules along the ventricle (Fig. 1). The nodules contain pyramidal neurons with immature morphology and haphazardly oriented apical dendrites, and without clear aggregation, as well as different subpopulations of GABAergic interneurons [4]. They mostly involve the trigone and occipital horn of lateral ventricle area, followed by the frontal and body parts, usually accompanied by ventriculomegaly and hydrocephalus. According to their distribution, five types of nodules can be distinguished: (a) bilateral and symmetrical; (b) bilateral single-noduled; (c) bilateral and asymmetrical; (d) unilateral; and (e) unilateral with extension to the neocortex (Fig. 2) [5]. The main clinical manifestations of PVNH are the trio of neurological dysfunction, intellectual disability (ID), and refractory epilepsy.

PVNH is a major disease that causes hereditary epilepsy, and 13−20% of patients with MCD and seizures have PVNH [6]. Seizures can strike as early as a few days after birth but are more common at 10–20 years old. Stereo-electroencephalogram (SEEG) recordings in patients with PVNH show the majority of seizures originate simultaneously from the nodules and the functionally related cortex, with others originating from the heterotopias alone [7]. Whole-brain high-resolution fiber tractography imaging indeed shows the presence of abnormal nerve fiber connections between ectopic nodules and the overlying cortex [8].

PVNH is known as a highly genetically heterogeneous disease, and recent studies emphasize novel single genetic mutation sites. These mutations may cause disruptions of microtubule or actin cytoskeleton organization, vesicle transport, cell adhesion, nucleus-cilia coupling, and other signaling pathways, thus impairing the proliferation, differentiation or migration of neuronal progenitor cells. We here review the risk genes with likely pathogenic changes that lead to PVNH in human patients, and present them chronologically by the time the first patient was reported, covering a period of more than two decades. For each of these genes, the physiological functions of its product are described, and the possible pathogenesis of these single genetic variants is also revealed.

## Risk genes sorted by the time of the first reported case

### *FLNA*

*FLNA* is the gene that is most closely associated with PVNH. Since PVNH was first mapped on *FLNA* in 1998 [9], multiple cases of *FLNA*-associated bilateral PVNH have been reported. Due to the X-linked dominant inheritance pattern, PVNH caused by *FLNA* variants occurs more commonly in female familial cases; because most male variant carriers do not survive in utero or in the first years of life. The manifestations include ID, developmental delay (DD), focal seizures, and extracranial complications like cardiac valvular dysplasia. *FLNA* (on Xq28) encodes filamin A, a widely expressed actin-binding protein that crosslinks actin filaments and membrane glycoproteins by interacting with integrins, transmembrane receptor complexes, and second messengers. This dimeric protein regulates remodeling of the actin cytoskeleton that is significant for the modulation of cell shape and migration. In *Flna* knockdown rats, the neuroepithelial lining of the brain is perturbed likely because of the disorganization of RGC apical complex integrity and polarized scaffolds [10]. *Flna* knockout male mouse models die at birth with severe vascular and cardiac defects [11], comparable with the pattern observed in human patients.

### *ARFGEF2*

Cases of autosomal recessive PVNH with microcephaly caused by *ARFGEF2* variants were first described by Sheen et al [12]. To date, 15 cases have been reported [12–16]. Phenotypes comprised microcephaly, epilepsy, quadriplegia and DD. Dystonia occurred occasionally [13, 14, 16], as well as recurrent infections, particularly in the respiratory tract [12, 14]. On MRI, cerebral atrophy and PVNH were observed in all patients, sometimes accompanied by basal ganglia and putamen hyperintensity, hippocampus and corpus callosum atrophy. *ARFGEF2* (on chr20q13.13) encodes brefeldin A-inhibited GEF2 protein (BIG2), a protein kinase A-anchoring protein highly expressed in neuronal progenitors. There is a physically coordinated link between BIG2 and ADP-ribosylation factor 1 (ARF1) to guide intracellular vesicle trafficking and assemble membrane proteins [17]. Inhibition of BIG2 in mice leads to abnormal transport of cell adhesion molecules (CAM), such as E-cadherin and β-catenin, from the Golgi apparatus to the cell membrane [12]. This disruption may impair neuroependymal integrity, radial glial scaffolding, as well as glial-guided neuronal migration [18]. An interaction between BIG2 and FLNA is reflected in *Arfgef2* knockdown mice, which show overexpression of phosphor-FLNA at Serine2152 and PVNH, where FLNA-actin binding affinity is weakened [19]. FLNA-BIG2 dependent endocytosis, moreover, reflects apical abscission at the last stage of cytokinesis, and its delay obstructs the migration of postmitotic neurons out of VZ [20].

### *LRP2*

Four siblings from consanguineous parents were found to carry the same homozygous mutation in *LRP2* [21]. All were diagnosed with Donnai-Barrow syndrome, characterized by large anterior fontanel, sensorineural deafness, diaphragmatic eventration, and proteinuria, and one sibling was determined to have PVNH, agenesis of the corpus callosum, disruption of frontal lobe development, and an abnormally placed central sulcus. *LRP2* (on chr2q31.1) encodes the lipoprotein receptor-related protein 2, also called megalin. LRP2 is a transmembrane endocytic receptor with a similar structure to the low-density lipoprotein receptor. It is found in the specialized epithelium of multiple organs, including the apical surface of the neuroepithelium in the developing brain [22]. A lack of LRP2 even disrupts the adult neurogenesis in the SVZ [23]. LRP2 is also significantly related to neural tube formation. It has been reported that LRP2 regulates apical constriction and appropriate orientation of a core planar cell polarity (PCP) protein required for neural tube closure [24]. In addition, *Lrp2*-mediated endocytosis enables metalloproteases to contribute to ciliogenesis in the neural tube and yolk sac [24]. All these influences in the nervous system are potential factors in the pathology of PVNH, although their exact role is uncertain.

### *ARX*

Cortical malformations caused by *ARX* mutants are commonly known as X-linked lissencephaly-2, however, PVNH was also identified in one case. A boy with anterior left PVNH had a 6-basepair insertion in *ARX* [25]. He experienced complex manifestations, such as DD, tonic spasms, axial hypotonia and spastic hemiplegia. MRI showed agenesis of the corpus callosum, ventricular dilatation, and polymicrogyria. *ARX* (on Xp21.3) encodes a highly conserved aristaless-related homeobox protein, a transcription factor responsible for brain morphogenesis. ARX is determined to maintain the length of the cell cycle and proliferation of progenitors [26]. *Arx* mutant mouse models developed periventricular accumulation of neurons by impaired acquirement of multipolar morphology, then reducing migration toward the cortex and disrupting late differentiation of *Arx* mutant cells in utero [26, 27].

### *DCHS1, FAT4*

Van Maldergem syndrome (VMS) involves mental retardation, renal hypoplasia, tracheal anomalies, auditory impairments, craniofacial abnormalities, malformed extremities, and PVNH that is often presents around the posterior horns of ventricles, accompanied by simplified cortical gyri of overlying regions. Nine VMS patients from seven pedigrees have been reported [28, 29]; seven patients had biallelic *DCHS1* or *FAT4* mutations, including three individuals and two pairs of siblings with PVNH ranging from bilateral nodular or laminar subependymal heterotopia to double cortex appearances. *FAT4* (on chr4q28.1) and *DCHS1* (on chr11p15.4) encode the receptor-ligand pair FAT4 and Dachsous1, both belonging to the protocadherin family and binding with each other to form an apically located adhesive complex that mediates apical anchoring of RGCs in the developing brain. Mouse models with *Fat4*^−/−^ or *Dchs1*^−/−^ result in a deficiency in cell junctions and the neuronal cytoskeleton, and subsequent migration capability and heterotopic accumulation [29]. Moreover, this knockout facilitates progenitor proliferation through mispositioning while inhibiting differentiation to neurons [30], suggesting that the combination of the migration block and the maintenance of progenitor proliferation likely underlies neuronal heterotopia [29]. In addition, *Dchs1* downregulation disrupts nucleus-cilia coupling in migrating neurons [31]. In addition to mouse models, cerebral organoids derived from induced pluripotent stem cells of patients carrying *FAT4* or *DCHS1* variants or from isogenic knockout lines, are utilized to replicate PVNH. The defective morphology of RGCs and changed migratory dynamics in a subset of neurons observed in these organoids, give rise to PVNH [32].

### *SHOC2*

In a previous report on Noonan syndrome, one patient with a *SHOC2* mutation had PVNH on the wall of the posterior right lateral ventricle [33]. In addition to typical Noonan syndrome and abnormalities outside the nervous system, the boy had DD, ID, hypotonia, and brisk deep tendon reflexes, and MRI showed ventriculomegaly, subarachnoid space enlargement, vertical tentorium and splenium of the corpus callosum. *SHOC2* (on chr10q25.2) encodes the protein with typical leucine-rich repeat domains through which it interacts with proteins with a variety of enzymatic activities. Zebrafish *shoc2* mutants are explored given that knockout of *Shoc2* in mouse models causes early embryonic lethality [34]. The SHOC2-ERK1/2 pathway is demonstrated to modulate cell–cell adhesion and collective cell migration [35]. In addition, SHOC2-depleted cells, in which neurons may be involved but without clear indications, affect cell attachment to matrix proteins and motility [36].

### *ERMARD*

There have been 17 patients with PVNH reported [37–41], whose clinical manifestations included ataxic gait, DD and facial dysmorphisms, harboring 6q25.3-q27 deletions of unequal sizes that shared a common minimal critical region containing candidate genes for brain structural abnormalities: *DLL1*, *PHF10*, *TCTE3*, *THBS2*, *WDR27* and *ERMARD*/*C6orf70* [41]. Two additional PVNH patients carried mosaic ring chromosome 6 with terminal deletions of 6q27, both presented with ID, microcephaly and developmental retardation [42, 43] similar to 6q terminal deletion syndrome. *ERMARD* (on chr6q27) encodes a plausible vesicular protein that is also an endoplasmic reticulum membrane-associated RNA degradation protein. In utero silencing of *Ermard* generates PVNH that is rescued by coexpression of ERMARD, while silencing of *Phf10* and *Dll1* only slightly delays neuronal migration, suggesting that haploinsufficiency or mutation of *ERMARD* might be the main cause of human PVNH [40]. Given the functions of contiguous genes of *ERMARD* associated with Notch signaling or neural stem cells [41], and the insufficient sample size of a single *ERMARD* variant, most studies still suggest that the combined haploinsufficiency of these candidate genes interferes with normal brain development.

### *SCN1A*

*SCN1A* (on chr2q24.3) encodes the α1 subunit of the voltage-gated sodium channel, essential for the generation and propagation of action potentials. Defects in this gene typically lead to Dravet syndrome featuring refractory seizures induced by fever in the first year of life after previously normal development. Brain imaging in most patients with *SCN1A* mutations is normal. However, three patients with MRI evidence of bilateral PVNH, exhibiting electroclinical features consistent with the Dravet syndrome spectrum, were determined to carry different *SCN1A* variants [44]. All patients had ataxia, ID of different degrees, and autistic features. One of them was also identified with right hemispheric dysplasia. Hence, although the variants may be rare from an epidemiological perspective, brain MRI should be assessed when *SCN1A*-associated epilepsy is suspected, particularly in individuals with a history of fever.

### *AKT3*

Megalencephaly-polymicrogyria with PVNH is a category of brain malformation syndrome caused by *AKT3* mutations. One patient, who suffered from refractory epilepsy, sustained hypoglycemia, generalized hypotonia, and DD, with enlarged ventricles and corpus callosum on MRI, harbored a germline *AKT3* missense variant that was demonstrated to activate *AKT3* signaling by in vitro experiments [45]. Another three cases with constitutional *AKT3* variants shared similar features, such as diffuse cortical dysplasia, bilateral PVNH, and ventriculomegaly [46]. *AKT3* (on chr1q44) encodes an isoform of AKT serine/threonine kinases that is highly expressed in the brain and acts as the most prominent AKT paralog during neurogenesis [47]. Despite being most associated with cancer, mutations in *AKT3* are also linked to an expanded spectrum of developmental brain disorders ranging from focal brain malformations such as hemimegalencephaly and polymicrogyria to diffuse bilateral cortical malformations, megalencephaly and heterotopia, determined by the type of mutation and degree of mosaicism [46]. *Akt3* mutant mouse models have neuronal heterotopias likely resulting from *Akt3* variant-dependent misexpression of reelin, a glycoprotein responsible for neuronal migration and lamination, in progenitors that leads to migration defects [48].

### *ARF1*

Three de novo, monoallelic missense *ARF1* variants have been reported in PVNH, with one patient presenting with delayed myelination, cortical thinning, and vermis atrophy on MRI. Limited language competence, epilepsy, brain dysgenesis, and developmental disability were also described [49]. *ARF1* (on chr1q42.13) encodes a Ras-superfamily GDP/GTP exchange protein directing COPI vesicle coat assembly and membrane transport by combining with both the membrane and the COPI heteroheptamer. ARF1 is also identified to regulate cytoskeleton reorganization by affecting actin dynamics [50]. Nucleotide-binding protein variants are predicted to cause rare diseases, and selective depletions of *ARF1* lay within the GTP/GDP-binding residues, affecting nucleotide activation, while depletions are not found in this region in the general population [49]. It is also suggested that loss of *ARF1*-activation functions occurs in *ARFGEF2*-associated PVNH, since *ARF1* is part of a signaling pathway with *FLNA* and *ARFGEF2*. However, because of embryonic lethality caused by a complete loss of *Arf1* function [51] and the limited number of reported cases of *ARF1*-associated PVNH, its pathogenic mechanism and connection with *ARFGEF2* are not widely accepted.

### *NEDD4L*

PVNH caused by *NEDD4L* mutations is accompanied by seizures, polymicrogyria, hypotonia, syndactyly, cleft palate, and DD, which is a novel hallmark to distinguish it from the *FLNA* variant. Fourteen cases of dominant heterozygous *NEDD4L* variants have been reported, targeting the HECT or WW domains respectively, which exhibited nearly all these consistent features [52–56]. *NEDD4L* (on chr18q21.31) encodes a HECT-type E3 ubiquitin ligase, a regulatory protein involved in mesenchymal differentiation and brain development. NEDD4L consists of a catalytic HECT domain and 2–4 WW domains. Most PVNH-associated variants are mapped to the HECT domain. This mutation adds to the sensitivity of the corresponding mutant to proteasome degradation, as amino acid alterations lead to conformation changes and subsequent catalytic activation that causes autoubiquitination and even degradation of the *NEDD4L* variant, as well as aberrant ubiquitination of *NEDD4L* substrates. These changes affect the orientation, proliferation and terminal translocation of neurons and progenitors through dysregulation of the AKT/mTOR and the TGF-β/Smad2/3 signaling pathways [52], which is thought to occur primarily during brain development [53]. Moreover, variants in the WW domain are predicted to disrupt protein–protein interactions and the recognition of substrates, and result in similar phenotypes to those caused by the HECT mutations.

### *GNAI2*

A de novo heterozygous missense mutation in *GNAI2* in an individual with PVNH was identified [57]. The patient had apparent ID, seizures and DD. MRI revealed ventricular enlargement and loss of white matter volume without microcephaly. The heterotrimeric guanine nucleotide-binding protein (G protein) is a signal transducer from G-protein-coupled receptors, and different combinations of αβγ subunits constitute specific G proteins. However, the α subunits are the most important, and *GNAI2* (on chr3p21.31) encodes the Gαi2 subunit. The Gi2 protein (Gαi2βγ5) is specifically localized in the VZ/SVZ and mediates the proliferation of neuronal progenitors and neurogenesis [58, 59]. The Gαi subunit is significant in regulating progenitor homeostasis by balancing self-renewal and differentiation in the developing brain [59]. Acute knockdown of *Gnai2* with in utero electroporation leads to a migration delay of cortical neurons toward the CP, but these excitatory neurons eventually reach the destination [57]. In the process of radial migration, characteristics of migration profiles and speed are found to be abnormal. However, deficiency of the Gαi2 subunit does not affect axon elongation, dendritic arbor formation or neurogenesis in the VZ, suggesting that the temporary delay itself possibly influences the formation of a normal neural network.

### *INTS8*

Compound heterozygous mutations in *INTS8* were identified in three siblings, who all exhibited neurodevelopmental disorders with PVNH, cerebellar hypoplasia, epilepsy, and spasticity [60]. The biallelic variants, both mapped to conserved regions, consisted of one missense mutation found in a splice site and one in-frame nine-base-pair deletion (*INTS8*ΔEVL for short) located within the fourth tetratricopeptide (TPR) domain. Analysis of patient cells and transfected cells indicated that the former variant led to altered splicing and mRNA decay, and *INTS8*ΔEVL disrupted the global integrity of the integrator complex (INT). The INT, which comprises 14 subunits, is linked to RNA polymerase II (RNAPII) and acts as a transcriptional switch to shape the transcriptome and ensure proper induction of genes. *INTS8* (on chr8q22.1) encodes INT subunit-8 that includes four TPR motifs which are involved in protein–protein interactions. Considering that *INTS8* contributes to the integrity and function of the entire INT [61], as demonstrated by decreased expression of other INT subunits in patient cells [60], *INTS8* mutations may impact the processes of transcription regulation. Moreover, introduction of the *INTS8*ΔEVL mutation in mouse P19 embryonic carcinoma cells perturbs retinoic acid-induced in vitro neuronal differentiation, as shown by the altered expression of differentiation markers [60]. Additionally, *INTS8* inhibits the formation of ectopic neuroblasts through interaction with the transcription factor Erm [62].

### *TMTC3*

Four siblings from a healthy family suffered from ID and recurrent nocturnal seizures, and three showed bilateral PVNH around the temporal horns on MRI [63]. All of them were identified to carry compound heterozygous *TMTC3* variants that were predicted to cause the loss of TPR domains and protein functions. *TMTC3* (on chr12q21.32) encodes a member of the transmembrane and tetratricopeptide repeat-containing proteins. TMTC3 is not only a member of a novel O-mannosylation pathway that specifically targets cadherin-like molecule processing [64], but also an O-mannosyl-transferase that enhances O-linked glycosylation and cadherin-mediated adherence [65]. The function of regulating O-mannosylation of E-cadherin and cell adhesion, whose association with PVNH remains unclear, has been utilized to study *TMTC3*-associated cobblestone lissencephaly [64]. In rat brains, TMTC3 colocalizes with a presynaptic marker for inhibitory synapses, indicating the involvement of *Tmtc3* in modulating GABAergic inhibitory synapses [63]. The listed interactors with TMTC in the BioGRID database, six in connection with ion channels and five oriented at the synapse [66], further support the possibility that neuronal migration might be accomplished by proper regulation of synaptic activity or Ca^2+^ gradients at GABAergic synaptic terminals [67].

### *MOB2*

A proband with bilateral PVNH presented with epilepsy and learning disabilities [31]. A compound heterozygous mutation in *MOB2* with strong evolutionary conservation was identified to be a loss-of-function (LoF) mutation. This mutation may reduce the expression of *MOB2* through nonsense mediated decay [68] or proteasome-dependent protein degradation. The family of monopolar spindle 1-binder (MOB) proteins constitutes highly conserved regulators of the Hippo signaling pathway. *MOB2* (on chr11q15.5) encodes MOB kinase activator 2 that interacts with the kinases NDR1/2. An extended distance between the nucleus and cilia and a deficiency in cilia number are observed in knockdown of *MOB2* respectively in the developing mouse cortex and in human cerebral organoids [31]. Given that the coupling of the cilia/centrosome and nucleus is significant for RGC-dependent neuronal migration [69], the deletion of *MOB2* disrupts these coordinated dynamics and, eventually, neuronal migration. Moreover, NDR1/2 bind to the C-terminus of MEKK1/2 kinase, where the most conserved sequence of MEKK family proteins is located, and the depletion of MEKK4 enhances FLNA phosphorylation and heterotopia formation [70]. Therefore, NDR1/2 potentially regulate MEKK4 activity, which is confirmed by increased FLNA phosphorylation at Serine2152 after *Mob2* knockdown [31].

### *KLHL7*

A homozygous in-frame deletion in *KLHL7* was identified in an individual [71]. The H-bond between Val61 and Ala65 was disrupted by the mutation, as was the α-helix, resulting in abnormal ubiquitination and accumulation of target substrates [72]. The boy was born with a Crisponi/CISS1-like phenotype and Bohring-Opitz-like syndrome, and also had DD, spasticity, and absent deep tendon reflexes. MRI revealed PVNH along the frontal horn of the right lateral ventricle, loss of supratentorial white matter volume, and corpus callosum atrophy. *KLHL7* (on chr7p15.3) encodes a Kelch family protein, and it is a substrate recognition component of the CUL3-based E3 ubiquitin ligase complex involved in substrate polyubiquitination for proteasome degradation. *KLHL7* probably participates in brain development, since malformations such as microcephaly and cortical dysplasia have been observed in other patients with *KLHL7* variants [73]. Mouse models show the expression of KLHL7 at E14.5 in the brain [74], but clear experiments to identify a specific link between KLHL7 and neurodevelopment are still lacking.

### *MAP1B*

*MAP1B* (on chr5q13.2) encodes microtubule-associated protein 1B that contains actin and microtubule binding domains, regulating the dynamics of both, and is mainly detected in the brain during the early stages of the neuronal development period [75]. MAP1B is a crucial component in cytoskeletal organization and axonal formation, thus guiding neuronal migration and development [76]. *MAP1B* LoF heterozygous variants were identified in four cases presenting with PVNH characterized by frontal-predominant nodules [77]. Interestingly, similar but milder neuroimaging findings, consisting of bilateral anterior PVNH and deep perisylvian and insular polymicrogyria, were found in one patient’s mother carrying the same variant. And a study of 31,463 individuals also identified three families with *MAP1B* variants, all of which were determined to produce truncated MAP1B contributing to PVNH, most of which caused ID [78]. Another *MAP1B* mutation in PVNH was reported, and this was the first study to describe relevant dysmorphic features in detail [75]. Despite recent discoveries of eight *MAP1B* mutants associated with PVNH, phenotypes ranging from clinical manifestations, such as ID, DD, seizures, and microcephaly to neuroimaging findings, such as corpus callosum and white matter volume, were variable across all of the patients, suggesting incomplete penetrance.

### *PLEKHG6*

*PLEKHG6* (on chr12p13.31) encodes the guanine nucleotide exchange factor MyoGEF that activates RhoA. RhoA, a small RhoGTPase whose conditional deficiency affects the RGC scaffold through alteration of actin and tubulin cytoskeleton stabilization, and causes distinct migration disorders, such as subcortical band heterotopia [79]. A proband with a homozygous mutation in *PLEKHG6* exhibited mild ID and bilateral PVNH in the trigone, posterior, and temporal horns of the lateral ventricles [80]. Epilepsy was not observed. This LoF variant is mapped to genomic elements related to the functions of bRGs, the expansion of which facilitates primate neocortical complexification [81]. Increased expression of the primate-specific isoform of *Plekhg6* via electroporation shows accumulation of ectopic neurons at the ventricular surface, as well as a defective apical adherent junction belt along the surface in cerebral organoids and alterations of the RGC scaffold in the developing mouse brain [80]. Moreover, in the mouse cortex, knockdown of *Plekhg6* gives rise to enhanced migration, even beyond the cortical plate, to form heterotopic clusters of neurons at the pial surface, which could be rescued by a construct encoding a fast-cycling form of RhoA [80].

### *KCNT1*

The co-occurrence of PVNH and *KCNT1* mutations in a proband with sleep-related frontal seizures was reported [82]. MRI showed left PVNH, and the nodule was connected by a radial band with the normal-appearing overlying cortex. She presented with severe psychosis, learning disabilities, and precocious puberty, which were also found in her younger brother who had the same mutation but FCD type Ib. *KCNT1* (on chr9q34.3) encodes a sodium-activated potassium channel that contributes to the slow hyperpolarization following repetitive firing in the nervous system. By modulating neuronal excitability through this pattern, mutations in this site are a topic of interest of studies related to epilepsy. Given previously reported MCD-associated ion channel gene mutations [44], and neuronal migration defects via an activity-dependent mechanism [83], mild structural changes in the brain can occur in conjunction with ion channel gene mutations, and contribute to seizures.

### *MEN1*

*MEN1* (on chr11q13.1) encodes menin, a nuclear scaffold protein that modifies histones, regulates epigenetic genes, and directs cell division. Menin binds to the p35 promoter region to promote p35 transcription and the p35-Cdk5 pathway that regulates neuronal microtubule dynamics [84]. Epilepsy and abnormal neuronal migration are observed in mouse models when Cdk5 is deficient [85]. Direct knockout of *Men1* results in embryonic lethality and neural tube nonclosure [86]. In fact, menin is a tumor suppressor linked to multiple endocrine neoplasia type 1 syndrome (MEN1 syndrome) that features neuroendocrine tumors. However, a proband with a mutation of *MEN1* showed bilateral posterior PVNH, refractory focal epilepsy, and morbid obesity without MEN1 syndrome [87]. This heterozygous variant, found in the brain sample extracted by SEEG electrodes but not in the blood, affects one of the two NLS domains that contribute to nuclear localization and subsequent transcriptional regulation. Because of the collected trace brain tissue that adheres to the electrodes in a single patient, reliable data about the true extent of mosaicism and comprehensive exon‐level deletion or duplication cannot be analyzed. Thus, quantitative identification of *MEN1* copy number variation is necessary.

### *ECE2*

*ECE2* (on chr3q27.1) encodes endothelin-converting enzyme 2, a metallopeptidase that is a rate-limiting enzyme catalyzing the production of endothelin vasoactive peptide family members. In a cohort of 202 probands [77], two were identified to carry biallelic missense variants in *ECE2* [88]. This study has also explored the pathogenic mechanism of *ECE2*. Knockdown of *ECE2* affects the morphology and polarity of apical radial glial cells (aRGs), such as the loss of apical attachment, leading to a delamination of progenitors and ectopic neurons. *ECE2* also stabilizes actin and microtubule cytoskeleton organization and the apical-basal polarity of aRGs by posttranslational modifications such as phosphorylation. These results suggest that *ECE2* supports neuronal migration by maintaining the RGC scaffold. Moreover, proteomic analysis indicates that extracellular matrix proteins are reduced predominantly when *ECE2* is knocked out, such as laminin, lumican and six different collagens, especially functional ones linked to cortical development. *ECE2* overexpression causes phenotypes similar to those observed following acute knockdown and rescues neuronal mispositioning through the latter, which indicates the significance of its proper expression for neuronal development and locomotion.

### *LAMC3*

A male fetus was diagnosed with extensive bilateral posterior PVNH, mainly involving the occipital horns, through postmortem MRI at 21 + 5 weeks gestation [89]. The aborted fetus had relatively normal growth parameters, and no additional malformations of brain structure were found. Compound heterozygous variants in *LAMC3* were confirmed in the proband. Similar to the previously reported pathogenic *LAMC3* mutation [90], the confirmed variants are located in the domain that is involved in the interaction with extracellular matrix molecules and the formation of basement membranes. *LAMC3* (on chr9q34.12) encodes the laminin subunit γ3. Laminins are a family of glycoproteins that are significant for cell adhesion, differentiation, and migration, and two out of three laminin isoforms composed of the γ3 subunit are only observed in the nervous system and are expressed in both the VZ and the CP, especially in the temporal and occipital lobes in the developing brain. *Lamc3* knockout mouse models reveal delayed migration and abnormal spatial patterning of astrocytes in the retina [91], and zebrafish models show perturbed migration of rostral primary motoneurons [92], indicating the important role of the laminin γ3 subunit in neuronal migration.

### *SYNCRIP*

*SYNCRIP* (on chr6q14.3) encodes a synaptotagmin‐binding cytoplasmic RNA‐interacting protein that regulates mRNA processing and maturation. SYNCRIP, also known as hnRNPQ, targets mRNAs which encode components of the microtubule network [93] and interact with RhoA that regulates actin dynamics related to neuronal morphology [94]. Thus, it potentially plays a causative role in neurodevelopmental disorders [95]. *SYNCRIP*-related PVNH is the most recently reported subtype. One individual with PVNH in the right frontal lobe and widened subarachnoid spaces showed myoclonic-atonic epilepsy with myoclonic reflex seizures caused in part by tactile stimuli, as well as ID and DD, and was determined to carry a *SYNCRIP* variant [96]. The severe variant probably leads to haploinsufficiency with the loss of RNA recognition motif domains that are highly vulnerable to variation and necessary for SYNCRIP to contact RNA. In contrast, N‐ and C‐terminal regions might be more flexible when mutated.

## Conclusions

This review comprehensively summarizes the risk genes that have already been clinically reported for PVNH. Although among these genes *FLNA* has been well studied, there is a lack of understanding of PVNH caused by other single-gene variants in clinical practice when considering relatively sporadic and rare diagnosis of this disease. In addition, human cerebral organoids and animal models can provide useful information or hypotheses that need to be verified in clinical practice. In consequence, relevant clinical manifestations and potential pathogenesis of PVNH associated with each risk gene are included here.

The distribution types of the nodules in PVNH may vary according to the clinically mutated risk gene (Fig. 2), but all the patients with PVNH suffer from complicated symptoms (Table 1). Intractable epilepsy is not always present [80] even if it was the most commonly observed. For most cases, in fact, seeking genetic backgrounds for PVNH, which involves exploratory mutation identifications for a potential locus in cohorts, is more helpful for the early intervention of diseases and provides directions for further basic research. In addition, neuronal migration is the most predominant at 12–24 weeks of gestation, indicating the potential role in identifying PVNH in pregnancy by ultrasound and MRI, which has been achieved in a fetus with *LAMC3* variants, whose parents chose to terminate the pregnancy at 21 + 5 weeks gestation according to the ultrasound results [89].

**Table 1:** Clinical manifestations and brain abnormalities of genetic changes

Gene	Inheritance	Human Locus	Main symptoms	Brain Abnormalities	References
*FLNA*	XLD	Xq28	DD, ID, Seizures, Extracranial complications	PVNH	Fox et al., 1998 [9]
*ARFGEF2*	AR	20q13.13	DD, Seizures, Dystonia	Microcephaly with PVNH, Hippocampus and corpus callosum atrophy	Sheen et al., 2004 [12] de Wit et al., 2009 [13] Tanyalçin et al., 2013 [14] Banne et al., 2013 [15] Bardón-Cancho et al., 2014 [16]
*LRP2*	AR	2q31.1	Proteinuria, Large anterior fontanel Sensorineural deafness	PVNH, Abnormally placed central sulcus, Agenesis of the corpus callosum, Disruption of frontal lobe development	Kantarci et al., 2007 [21]
*ARX*	XLR	Xp21.3	DD, Tonic spasms, Axial hypotonia, Spastic hemiplegia	Polymicrogyria, Ventricular dilatation, Agenesis of corpus callosum	Oegema et al., 2012 [25]
*DCHS1* *FAT4*	AR	11p15.4 4q28.1	Van Maldergem syndrome	PVNH with overlying simplified cortical gyri	Mansour et al., 2012 [28] Cappello et al., 2013 [29]
*SHOC2*	AD	10q25.2	DD, ID, Hypotonia, Brisk deep tendon reflexes	PVNH, Ventriculomegaly, Subarachnoid space enlargement, Vertical splenium of the corpus callosum	Gripp et al., 2013 [33]
*ERMARD*	AD	6q27	DD, ID, Seizures	Microcephaly with PVNH, Hydrocephalus, Corpus callosum dysgenesis	Conti et al., 2013 [40] Peddibhotla et al., 2014 [41]
*SCN1A*	AD	2q24.3	ID, Ataxia, Seizures, Autistic features	PVNH	Barba et al., 2014 [44]
*AKT3*	AD	1q44	DD, Seizures, Hypotonia, Infantile spasms	Megalencephaly-Polymicrogyria with PVNH, Ventriculomegaly, Cortical dysplasia	Nellist et al., 2015 [45] Alcantara et al., 2017 [46]
*ARF1*	AD	1q42.13	DD, Seizures, Limited language competence	PVNH, Brain dysgenesis	Ge et al., 2016 [49]
*NEDD4L*	AD	18q21.31	DD, Seizures, Hypotonia	PVNH, Polymicrogyria	Broix et al., 2016 [52] Kato et al., 2017 [53] Elbracht et al., 2018 [54] Kato et al., 2019 [55] Stouffs et al., 2020 [56]
*GNAI2*	AD	3p21.31	DD, ID, Seizures	PVNH, Ventricular enlargement, Volume loss of white matter	Hamada et al., 2017 [57]
*INTS8*	AR	8q22.1	ID, Seizures, Facial and limb dysmorphism	PVNH, Cerebellar hypoplasia	Barsh et al., 2017 [60]
*TMTC3*	AR	12q21.32	ID, Nocturnal seizures	PVNH	Farhan et al., 2017 [63]
*MOB2*	AD	11p15.5	Seizures, Learning disability	PVNH	O’Neill et al., 2018 [31]
*KLHL7*	AR	7p15.3	DD, Spasticity, Absent deep tendon reflexes	PVNH, Corpus callosum atrophy, Volume loss of white matters	Kanthi et al., 2019 [71]
*MAP1B*	AD	5q13.2	DD, ID, Seizures	Microcephaly with PVNH, Polymicrogyria, Corpus callosum dysgenesis	Hallmayer et al., 2018 [77] Walters et al., 2018 [78] Julca et al., 2019 [75]
*PLEKHG6*	AR	12p13.31	ID	PVNH	O'Neill et al., 2018 [80]
*KCNT1*	AD	9q34.3	Psychosis, Learning disabilities, Sleep-related frontal seizures	PVNH	Rubboli et al., 2019 [82]
*MEN1*	AD	11q13.1	Seizures, Morbid obesity	PVNH	Montier et al., 2019 [87]
*ECE2*	AR	3q27.1	No data	PVNH	Buchsbaum et al., 2020 [88]
*LAMC3*	AR	9q34.12	No data	PVNH	De Angelis et al., 2021 [89]
*SYNCRIP*	AD	6q14.3	DD, ID, Myoclonic-atonic epilepsy	PVNH	Semino et al., 2021 [96]

*PVNH* periventricular nodular heterotopia, *AD* autosomal dominant, *AR* autosomal recessive, *XLD* X-linked dominant, *XLR* X-linked recessive, *DD* development delay, *ID* intellectual disability

Rodent models are the most commonly used to study PVNH (Table 2). Neuronal migration lasts for several weeks and even continues after birth for better integration into neural circuits [97]. Most studies have emphasized the direct abnormalities of the migration process, such as in *FLNA*, *DCHS1*, *FAT4*, *GNAI2*, *MOB2*, *MAP1B*, *PLEKHG6*, *ECE2*, which are related to RGC morphology, cytoskeleton, adhesion, apical anchoring, and nucleus-cilia coupling. Nevertheless, additional causes of heterotopia have been increasingly realized (Table 3). For instance, vesicle trafficking as well as membrane transport, like in *ARFGEF2*, *ARF1*, and *ERMARD*, are also regarded to influence cortical development. Voltage-gated ion channel-encoding genes, considered as hotpot mutation sites of epilepsy, such as *SCN1A*, *KCNT1*, might also contribute to the formation of MCD. Given that a great deal of studies have confirmed that PVNH has a potential correlation with specific single-gene mutations that contribute to the above causative mechanisms, it is necessary for patients with PVNH to complete exome analysis for a more proper treatment recommendation and reassurance in further pregnancies.

**Table 2:** Observations of rodent models simulating genetic defects

Gene	Rodent models	Main observations	References
*Flna*	knockdown knockout (male)	PVNH and disrupted neuroepithelial lining Embryonic lethality with vascular and cardiac defects	Carabalona et al.,2012 [10] Feng et al., 2006 [11]
*Arfgef2*	knockdown	PVNH and impaired neuronal migration	Zhang et al., 2012 [19]
*Lrp2*	knockdown	Reduced vesicles at the cilium base	Kowalczyk et al., 2021 [24]
*Arx*	mutant	Periventricular neuronal accumulation, Reduced neuronal migration	Friocourt et al., 2008 [26] Colombo et al., 2007 [27]
*Dchs1* *Fat4*	knockout	Neuronal heterotopia within the subcortex, Maintenance of progenitor proliferation while mispositioning	Cappello et al., 2013 [29]
*Shoc2*	knockout	Embryonic lethality	Jang et al., 2019 [34]
*Ermard*	knockdown	PVNH	Conti et al., 2013 [40]
*Scn1a*	N/A	N/A	N/A
*Akt3*	mutant	Neuronal heterotopia	Baek et al., 2015 [48]
*Arf1*	knockout	Embryonic lethality	Hayakawa et al., 2014 [51]
*Nedd4l*	mutant	Neuronal migration and positioning defects	Broix et al., 2016 [52]
*Gnai2*	knockdown	Delayed radial migration	Hamada et al., 2017 [57]
*Ints8*	*INTS8*ΔEVL	Perturbation of neuronal differentiation	Barsh et al., 2017 [60]
*Tmtc3*	N/A	N/A	N/A
*Mob2*	knockdown	Neuronal heterotopia, Extended distance between the nucleus and cilia	O’Neill et al., 2018 [31]
*Klhl7*	N/A	N/A	N/A
*Map1b*	deficient	Neuronal migration defect	Del Río et al., 2004 [76]
*Plekhg6*	knockdown	Heterotopic neuronal clusters at the pial surface	O'Neill et al., 2018 [80]
*Kcnt1*	N/A	N/A	N/A
*Men1*	knockout	Embryonic lethality	Bertolino et al., 2003 [86]
*Ece2*	knockdown	Neuronal heterotopia	Buchsbaum et al., 2020 [88]
*Lamc3*	knockout	Delayed neuronal migration	Gnanaguru et al., 2013 [91]
*Syncrip*	N/A	N/A	N/A

**Table 3:** Potential mechanisms by which genetic changes lead to heterotopia

Gene	Encoding proteins	Ectopic mechanisms	References
*FLNA*	An actin-binding protein	Impaired remodeling of the actin cytoskeleton	Fox et al., 1998 [9]
*ARFGEF2*	A protein kinase A-anchoring protein	Impaired membrane proteins assembly, Overexpression of phosphor-FlnA at Serine2152, Disrupted ependymal integrity and apical abscission	Sheen et al., 2004 [12] Zhang et al., 2013 [17] Zhang et al., 2012 [19] Sheen et al., 2014 [20]
*LRP2*	A lipoprotein receptor-related endocytic receptor	N/A	N/A
*ARX*	An Aristaless-related homeobox transcription factor	Impaired acquirement of multipolar morphology	Friocourt et al., 2008 [26] Colombo et al., 2007 [27]
*DCHS1* *FAT4*	Receptor-ligand pair proteins of the protocadherin family	Deficiency in cell junction or neuronal cytoskeleton, Maintenance of progenitor proliferation, Defective morphology of RGCs, Impaired nucleus-cilia coupling	Cappello et al., 2013 [29] Roy et al., 2019 [30] O’Neill et al., 2018 [31] Klaus et al., 2019 [32]
*SHOC2*	A leucine-rich repeat protein	Affected cell–cell adhesion and mobility	Kota et al., 2019 [35] Jeoung et al., 2016 [36]
*ERMARD*	An endoplasmic reticulum membrane-associated RNA degradation protein	Impaired vesicular trafficking	Conti et al., 2013 [40]
*SCN1A*	The α1 subunit of sodium voltage-gated channel	N/A	N/A
*AKT3*	An isoform of AKT serine/threonine kinases	N/A	N/A
*ARF1*	A Ras-superfamily GDP/GTP exchange protein	Impaired cytoskeleton reorganization	Rocca et al., 2013 [50]
*NEDD4L*	A HECT-type E3 ubiquitin ligase	Dysregulation of AKT/mTOR and TGF-β/Smad2/3 signaling pathways	Broix et al., 2016 [52]
*GNAI2*	The α subunit of G protein	Abnormal neuronal morphology	Hamada et al., 2017 [57]
*INTS8*	A subunit of the integrator complex	Perturbed interaction with Erm	Zhang et al., 2019 [62]
*TMTC3*	An O-mannosyl-transferase	Impaired cell–cell adhesion	Larsen et al., 2017 [64]
*MOB2*	A monopolar spindle-one-binder kinase activator	Impaired nucleus-cilia coupling, Overexpression of phosphor-FlnA at Serine2152	O’Neill et al., 2018 [31]
*KLHL7*	A substrate recognition subunit of the E3 ubiquitin ligase complex	N/A	N/A
*MAP1B*	A microtubule associated protein	Disrupted cytoskeletal organization	Del Río et al., 2004 [76]
*PLEKHG6*	A guanine nucleotide exchange factor	Impaired RGC scaffold, Defective apical adherent junction belt	O'Neill et al., 2018 [80]
*KCNT1*	A sodium-activated potassium channel	N/A	N/A
*MEN1*	A nuclear scaffold protein	Dysregulation of neuronal microtubules dynamics	Shah et al., 2017 [84]
*ECE2*	An endothelin-converting enzyme	Impaired RGC morphology and polarity, Reduced secretion of extracellular matrix proteins	Buchsbaum et al., 2020 [88]
*LAMC3*	The γ3 of subunit laminin	Impaired cell–cell adhesion	Gnanaguru et al., 2013 [91]
*SYNCRIP*	A synaptotagmin‐binding cytoplasmic RNA‐interacting protein	Destabilization of microtubule network, Impaired actin dynamics	Khudayberdiev et al., 2021 [93] Xing et al., 2012 [94]

However, differences in species evolution between humans and rodents pose challenges for accurately mapping these findings in basic research to the development of the human brain. Except for knockdown of only a few genes, such as *Arfgef2* [19], *Ermard* [40], generating PVNH, a well-matched phenotype to human patients is difficult to observe in animal models with genetic intervention. Interestingly, *Plekhg6* knockdown mouse models even exhibit cobblestone lissencephaly [80], a type of MCD with excessive neuronal migration, as opposed to the pathology of PVNH. Complete knockout of *Arfgef2*, *Arf1*, *Lrp2*, and *Men1* can result in the most severe presentation of embryonic lethality. These different presentations are probably due to the variation in mouse bRGs compared to human bRGs that are highly proliferative and particularly abundant [98]; human gene expression patterns and complexity that are superior in the cortical germinal zones, particularly in the SVZ [99]; or differences at the genetic level. In contrast, human cerebral organoids that have gradually appeared in recent studies, such as *PLEKHG6, ECE2* modulation organoids, avoid confounding variables introduced in animal models and are more consistent with clinical manifestations.

Notably, targeted therapies for PVNH remain lacking, but recent advances in activity-dependent gene therapy offer promising insights. A cell-autonomous strategy was reported [100] using the c-Fos promoter to drive Kv1.1 potassium channel expression, which selectively modulated hyperactive neurons in epilepsy models without disrupting normal function. Given that PVNH, caused by monogenic mutations, usually presents with epileptic comorbidities due to abnormal neurons, this on-demand gene therapy paradigm provides a valuable framework. It highlights the potential of targeting pathogenic neuronal activity in PVNH-associated genetic defects, paving the way for future tailored therapies.

In conclusion, an updated and comprehensive spectrum of PVNH-associated risk genes is provided here, encompassing 23 genes with diverse inheritance patterns. We hope that in addition to deepening insights into the genetic architecture underlying neurodevelopmental processes, this review offers a targeted reference for clinicians evaluating patients with PVNH and guides future genetic screening efforts in larger PVNH cohorts.

## Data Availability

Research and review articles from reputed journals of high impact were taken and the information gathered from them was compiled into this review article manuscript. All the authors collectively created the figures and tables in this review article.

## Abbreviations

PVNH: Periventricular nodular heterotopia
MCD: Malformation of cortical development
NECs: Neuroepithelial cells
VZ: Ventricular zone
RGCs: Radial glial cells
IZ: Intermediate zone
CP: Cortical plate
IPCs: Intermediate progenitors
bRGs: Basal radial glial cells
SVZ: Subventricular zone
GBH: Gray matter heterotopia
ID: Intellectual disability
SEEG: Stereo-electroencephalogram
DD: Developmental delay
VMS: Van Maldergem syndrome
G protein: Guanine nucleotide-binding protein
TPR: Tetratricopeptide domain
INT: Integrator complex
RNAPII: RNA polymerase II
LoF: Loss-of-function
MOB: Monopolar spindle 1-binder
MEN1 syndrome: Multiple endocrine neoplasia type 1 syndrome
aRGs: Apical radial glial cells
